# Prevalence of *Aspergillus fumigatus* skin positivity in adults without an apparent/known atopic disease in Uganda

**DOI:** 10.1177/20499361211039040

**Published:** 2021-08-12

**Authors:** Richard Kwizera, Felix Bongomin, Ronald Olum, William Worodria, Freddie Bwanga, David B. Meya, Bruce J. Kirenga, Robin Gore, Stephen J. Fowler, David W. Denning

**Affiliations:** Department of Research, Infectious Diseases Institute, College of Health Sciences, Makerere University, P.O. BOX 22418, Kampala, Central, Uganda,Makerere University Lung Institute, College of Health Sciences, Makerere University, Kampala, Uganda; Department of Medical Microbiology, Faculty of Medicine, Gulu University, Gulu, Uganda, Department of Medicine, School of Medicine, College of Health Sciences, Makerere University, Kampala, Uganda; Department of Medicine, School of Medicine, College of Health Sciences, Makerere University, Kampala, Uganda; Department of Medicine, School of Medicine, College of Health Sciences, Makerere University, Kampala, Uganda, Division of Pulmonology, Mulago National Referral Hospital, Kampala, Uganda; Department of Medical Microbiology, School of Biomedical Sciences, College of Health Sciences, Makerere University Kampala, Uganda; Infectious Diseases Institute, College of Health Sciences, Makerere University, Kampala, Uganda, Department of Medicine, School of Medicine, College of Health Sciences, Makerere University, Kampala, Uganda; Makerere University Lung Institute, College of Health Sciences, Makerere University, Kampala, Uganda, Department of Medicine, School of Medicine, College of Health Sciences, Makerere University, Kampala, Uganda, Division of Pulmonology, Mulago National Referral Hospital, Kampala, Uganda; Cambridge University Hospitals NHS Foundation Trust, Cambridge, Cambridgeshire, UK; Division of Infection, Immunity and Respiratory Medicine, School of Biological Sciences, Faculty of Biology, Medicine and Health, The University of Manchester, NIHR Biomedical Research Centre, Manchester University Hospitals NHS Foundation Trust, UK; Division of Infection, Immunity and Respiratory Medicine, School of Biological Sciences, Faculty of Biology, Medicine and Health, The University of Manchester, NIHR Biomedical Research Centre, Manchester University Hospitals NHS Foundation Trust, UK

**Keywords:** *Aspergillus* sensitisation, atopy, fungal allergy, skin prick testing, Uganda

## Abstract

**Background:**

Skin prick testing (SPT) is an important investigation in the evaluation of allergy to fungal pathogens. However, the background sensitivity to fungal allergens among healthy people in Uganda is unknown. Our aim was to assess the background prevalence of *Aspergillus fumigatus* SPT positivity in apparently healthy adults without known atopic disease in Uganda.

**Methods:**

For this pilot study, we recruited 50 healthy volunteers using convenience sampling, 56% of whom were health workers. We performed the SPT for *A. fumigatus* according to manufacturer’s instructions. A wheal diameter of ⩾3 mm was considered positive.

**Results:**

The prevalence of *A. fumigatus* skin positivity was 60% (30/50). Participants with a positive *A. fumigatus* SPT were significantly younger than those with a negative result [median age (years): 28 *versus* 35; *p* = 0.005].

**Conclusion:**

There is a high skin positivity against *A. fumigatus* among non-atopic healthy Ugandan adults. There is an urgent need to establish a normal wheal cut-off value for this population. SPT alone may be an unreliable test for the diagnosis of *A. fumigatus* associated allergic syndromes. More studies are needed to define the prevalence of *A. fumigatus* skin positivity among non-atopic healthy population in Africa.

## Introduction

Fungal allergy in the context of asthma,^1^ non-cystic fibrosis bronchiectasis,^2^ tuberculosis,^3^ chronic obstructive pulmonary disease (COPD),^4^ or fungal rhinosinusitis is one of the most common health problems globally.^5^ However, the pathogenic significance of fungi from the genera *Alternaria*, *Cladosporium*, *Penicillium* and *Aspergillus* is poorly described in Africa.^6^ The burden of serious fungal diseases in Uganda is high, with an estimated 2.5 million cases per year,^7^ yet the index of clinical suspicion remains low,^8^ despite recent evidence showing that *Aspergillus* species are significant causes of morbidity in different at-risk populations in Uganda.^3,6–10^

The lungs are the primary site of infection by *Aspergillus* species, causing disorders including allergic, chronic, sub-acute and invasive pulmonary aspergillosis,^11^ with *Aspergillus fumigatus* the most commonly implicated species.^12^ Human allergic disorders associated with *A. fumigatus* include allergic asthma, severe asthma with fungal sensitisation (SAFS), allergic rhinosinusitis, allergic bronchopulmonary aspergillosis (ABPA), and hypersensitivity pneumonitis.^13^ In addition, *A. fumigatus* is isolated frequently from the respiratory tract of patients with asthma who do not meet the criteria for ABPA or SAFS, and occasionally in the respiratory tract of healthy individuals.^14^ Diagnosis of *Aspergillus* sensitization requires demonstration of evidence of allergic sensitization to *Aspergillus* either by skin prick testing (SPT) or *Aspergillus*-specific IgE immunoassays.^1^

There is a paucity of data on the frequency of SPT reactivity against *A. fumigatus* in healthy adults without known atopic disease. We therefore aimed to describe the distribution of *A. fumigatus* SPT positivity among healthy adults without known atopic disease in Uganda, which will, in turn, generate hypotheses to encourage further research in this field of fungal allergy in Uganda.

## Methods

This was a cross-sectional study evaluating the frequency of *A. fumigatus* skin prick positivity among healthy adults without known atopic disease in Uganda. It was carried out between March and October 2019. This study was nested within the African Severe Asthma Program (ASAP) clinical study [ClinicalTrials.gov identifier: NCT03065920] at the Makerere University Lung Institute.^15^ ASAP is a clinical study with the primary objective being to identify and characterize severe asthma in Uganda, Kenya, and Ethiopia. Participants provided written informed consent to participate in this study. Ethics approval for this sub-study was obtained from the School of Biomedical Sciences Research and Ethics Committee (SBS 598), the Uganda National Council for Science and Technology (HS 2532), and the Uganda National Drug Authority (9464). All participants in the current study were healthy adults (⩾18 years), without known atopic disease or respiratory conditions, and not taking steroids or oral antihistamines for any reason in the last 7 days. Since there is limited literature on the prevalence of *Aspergillus* skin positivity among healthy population, to understand prevalence of *Aspergillus* sensitisation in Ugandan healthy population, we consented and tested healthy volunteers. We used healthy individuals from the general population, including medical workers, medical students, and support staff at Makerere University and Kiruddu National Referral Hospital. A convenience sampling method was employed for this pilot study.

*A. fumigatus* SPT [Immunospec (Pty) Ltd, Johannesburg, Gauteng, South Africa] was performed and the results interpreted according to international guidelines.^16^ Normal saline served as a negative control while histamine was the positive control, with a mean wheal diameter of at least 3 mm being considered positive, after 15 min of allergen application. We did not perform testing for total serum or *A. fumigatus*-specific IgE.

Data were analyzed using STATA® version 16 (STATA, College Station, TX, USA). Primary data analysis aimed to describe the distribution of *A. fumigatus* skin prick positivity at a 95% confidence interval (CI).

## Results

Between March and October 2019, we enrolled 50 eligible participants, of whom 28 (56%) were female; the median age for all participants was 30 years [interquartile range (IQR) = 27–35). A total of 28 (56%) of the participants were health workers (Table 1).

**Table 1.** Baseline characteristics of the study population.

Demographics	Frequency (%)
Age, median (IQR)	30 (27–35)
Female, *n* (%)	28 (56)
Occupation, *n* (%)	
Health worker	28 (56)
Volunteer	5 (10)
Counsellor	4 (8)
Records officer	4 (8)
Student	4 (8)
Information technology specialist	3 (6)
Self employed	1 (2)
Project administrator	1 (2)

IQR, interquartile range.

**Figure 1.**
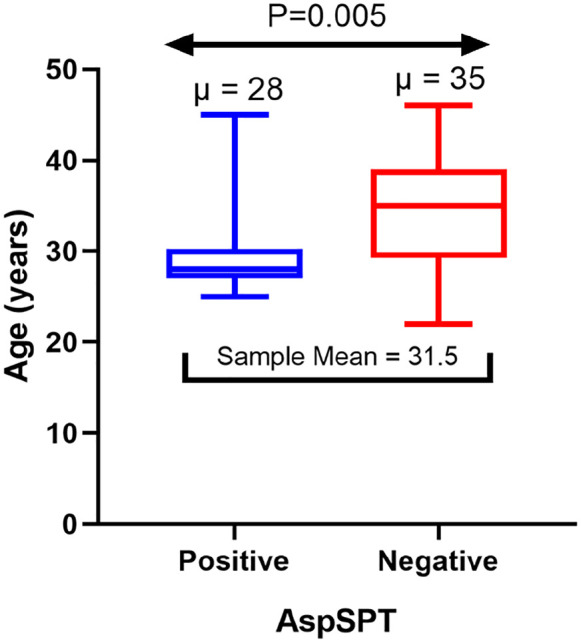
Distribution of age by *Aspergillus fumigatus* skin positivity. AspSPT, *A. fumigatus* skin prick test.

**Figure 2.**
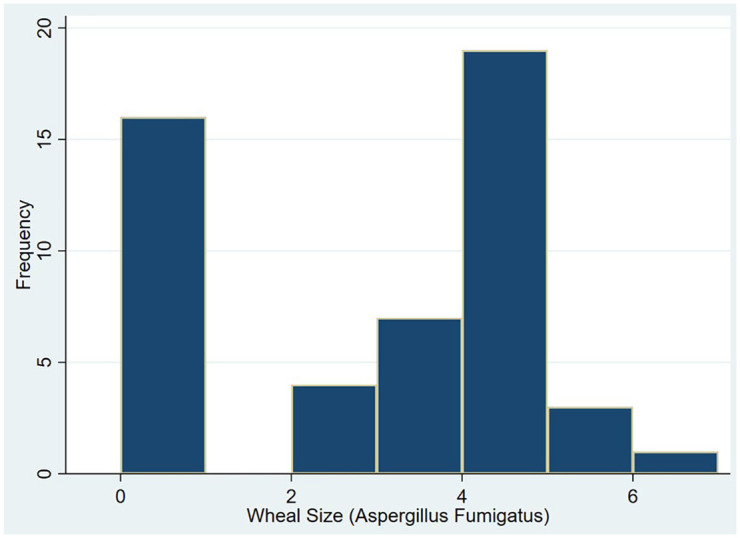
Distribution of wheal size for *Aspergillus fumigatus*. Wheal size (µ) is measured in millimeters.

The prevalence of *A. fumigatus* skin positivity was 60% (30/50) (95% CI = 45.6–72.8). There was a significant difference in age between participants who were positive for *A. fumigatus* skin test and those who were negative (*p* = 0.005) (Figure 1). There was no significant difference in *A. fumigatus* reactivity by gender (*p* = 0.907) or occupation (*p* = 0.612). None of the participants reacted to the negative control. Only one participant had a wheal diameter of 2 mm and the rest had zero against the negative control. Figure 2 is a histogram of wheal sizes for *A. fumigatus* skin prick test for this population.

## Discussion

In the present study, we report a high prevalence (60%) of *A. fumigatus* skin positivity among healthy adults without known atopic disease in Uganda. This could indicate the fact that apparently healthy people have an undiagnosed fungal atopy. Alternatively, this could also indicate that *A. fumigatus* is the commonest fungal allergen in Uganda, similar to what is reported in the developed countries. However, a study from India showed a 2% prevalence of *A. fumigatus* skin positivity among healthy controls.^17^ In addition, our previous work among adult asthmatics in Uganda showed a lower prevalence (48%) of *A. fumigatus* skin reactivity than the 60% observed in this study despite the atopic nature of asthmatics.^10^ We cannot rule out the possibility of cross-reactivity to *A. fumigatus* crude extracts, which often do not indicate genuine sensitization.^13,18^ Besides, products secreted after conidial germination into hyphae can be differentially recognized by protective T-cells in healthy non-atopic individuals.^19^ Therefore, SPT should not be used alone to diagnose *A. fumigatus* sensitivity in Uganda since the background prevalence in healthy populations is apparently very high based on the results of this pilot study. However, we recommend that larger studies be carried out to confirm these data. The *in vitro A. fumigatus-*specific IgE may be more informative to reflect the actual burden. Those with skin positivity were significantly younger. However, the reason for this observation is unclear and not really clinically relevant.

Our study is limited by its very small sample size and sampling frame, which limits its generalization to the general population. The optimal cut-off wheal size to define *A. fumigatus* in our population is unknown. This could have led to an over-estimation of the prevalence of *A. fumigatus* SPT reactivity in this population. One uncertainty is whether the 3 mm cut-off is appropriate for Uganda; a 5 mm cut-off would reduce the prevalence of *A. fumigatus* sensitisation to 8%, which is more reasonable. Therefore, we propose to define a suitable cut-off wheal size in healthy adults. However, our estimate provides baseline information to encourage further research in the field of fungal allergy in Africa. More studies are needed to define the prevalence of *A. fumigatus* skin positivity among non-atopic healthy population in Africa. There is an urgent need to establish a normal wheal cut-off value for this population.

## Conclusion

In conclusion, we found a high prevalence of *A. fumigatus* skin positivity in apparently healthy non-atopic individuals in Uganda. Those with skin positivity were significantly younger. Given a very high background *A. fumigatus* skin positivity rate in our setting, SPT alone may be an unreliable test for the diagnosis of *A. fumigatus*-associated allergic syndromes. We propose to define a suitable cut-off wheal size in healthy adults.

## Supplemental Material

sj-xlsx-1-tai-10.1177_20499361211039040 – Supplemental material for Prevalence of Aspergillus fumigatus skin positivity in adults without an apparent/known atopic disease in Uganda Click here for additional data file. Supplemental material, sj-xlsx-1-tai-10.1177_20499361211039040 for Prevalence of Aspergillus fumigatus skin positivity in adults without an apparent/known atopic disease in Uganda by Richard Kwizera, Felix Bongomin, Ronald Olum, William Worodria, Freddie Bwanga, David B. Meya, Bruce J. Kirenga, Robin Gore, Stephen J. Fowler and David W. Denning in Therapeutic Advances in Infectious Disease
